# Current status of cardiac regenerative medicine; An update on point of view to cell therapy application

**DOI:** 10.34172/jcvtr.2020.44

**Published:** 2020-11-24

**Authors:** Mehdi Hassanpour, Nasser Aghamohamadzade, Omid Cheraghi, Morteza Heidarzadeh, Mohammad Nouri

**Affiliations:** ^1^Stem Cell Research Center, Tabriz University of Medical Sciences, Tabriz, Iran; ^2^Department of Clinical Biochemistry and Laboratory Medicine, Tabriz University of Medical Sciences, Tabriz, Iran; ^3^Stem Cell and Regenerative Medicine Institute, Tabriz University of Medical Sciences, Tabriz, Iran; ^4^Endocrine Research Center, Tabriz University of Medical Sciences, Tabriz, Iran; ^5^Department of Biochemistry, Faculty of Biological Science, Tarbiat Modares University, Tehran, Iran; ^6^Research Center for Translational Medicine, Istanbul, Turkey

**Keywords:** Cardiovascular Diseases, Acute Myocardial Infarction, Regenerative Medicine, Cell Therapy

## Abstract

Cardiovascular diseases (CVDs) are the leading cause of death globally. Because of the economic and social burden of acute myocardial infarction and its chronic consequences in surviving patients, understanding the pathophysiology of myocardial infarction injury is a major priority for cardiovascular research. MI is defined as cardiomyocytes death caused by an ischemic that resulted from the apoptosis, necrosis, necroptosis, and autophagy. The phases of normal repair following MI including inflammatory, proliferation, and maturation. Normal repair is slow and inefficient generally so that other treatments are required. Because of difficulties, outcomes, and backwashes of traditional therapies including coronary artery bypass grafting, balloon angioplasty, heart transplantation, and artificial heart operations, the novel strategy in the treatment of MI, cell therapy, was newly emerged. In cell therapy, a new population of cells has created that substitute with damaged cells. Different types of stem cell and progenitor cells have been shown to improve cardiac function through various mechanisms, including the formation of new myocytes, endothelial cells, and vascular smooth muscle cells. Bone marrow- and/or adipose tissue-derived mesenchymal stem cells, embryonic stem cells, autologous skeletal myoblasts, induced pluripotent stem cells, endothelial progenitor cells, cardiac progenitor cells and cardiac pericytes considered as a source for cell therapy. In this study, we focused on the point of view of the cell sources.

## Introduction

Based on WHO updates in May 2017, cardiovascular diseases (CVDs) are the leading cause of death globally. An estimated 17.7 million people died from CVDs in 2015, representing 31% of all global deaths. Of these deaths, an estimated 7.4 million were due to coronary heart disease and 6.7 million were due to stroke. There are 32.4 million Acute Myocardial Infarction (AMI) worldwide every year. Over three-quarters of CVD deaths take place in low- and middle-income countries. People with cardiovascular disease or who are at high cardiovascular risk (due to the presence of one or more risk factors such as hypertension, diabetes, hyperlipidemia, or already established disease) need early detection and management using counseling and medicines.^1^ Because of the high socioeconomic burden of AMI and its chronic consequences in surviving patients, understanding the pathophysiology of infarcted cardiac injury is a major priority for cardiovascular research.

### Symptoms of heart attacks

A heart attack or MI may be the first warning of an underlying disease. Symptoms of a heart attack include: discomfort in the center of the chest, arms, left shoulder, elbows, jaw, or back; numbness of the face, arm, or leg, especially on one side of the body; Palpitations (feeling like your heart is beating too fast or irregularly); confusion, difficulty speaking or understanding speech; nausea or vomiting; shortness of breath; Sweating and finally, fainting or unconsciousness.^2^

### Criteria for AMI

From point of view of Joint European Society of Cardiology (ESC), the American College of Cardiology Foundation (ACCF), the American Heart Association (AHA) and the World Heart Federation (WHF) very small amounts of myocardial injury or necrosis that can be detected by biochemical markers and/or imaging, is called MI. Under these conditions, one of the following criteria meets the diagnosis of MI: Rise of cardiac biomarkers such as cardiac troponin I (cTnI) and creatine kinase (CK); vicissitude in ECG such as ST elevation and development of Q waves; Imaging evidence of loss of viable myocardium;^3^ Symptoms of ischemia and Identification of an intracoronary thrombus by angiography.^4^

### Pathophysiology of MI

From a pathologic perspective, MI is defined as cardiomyocytes (CMs) death caused by an ischemic insult. The close spatial association between leukocytes and viable CMs in the border zone and the injurious potential of subsets of blood-derived cells generated the concept of leukocyte-mediated CM injury. Neutrophils interact with endothelial cells (ECs), roll along the endothelial surface, decelerate to a firm arrest, transmigrate across the vascular wall, infiltrate the infarct, and adhere to viable CM exerting cytotoxic effects and extending ischemic injury. Infiltrating leukocytes are also important for infarct repair by releasing proteases and reactive oxygen specious, thereby clearing the wound from dead cells and debris.^5^

The role of the chemokines in the recruitment of leukocyte subpopulations in the infarcted heart has validated in recent studies. Both CXC and CC chemokines are upregulated in the infarcted heart, bind to glycosaminoglycans on the endothelial surface and interact with chemokine receptors expressed by leukocytes. CCL2/CCR2 interactions are implicated in the recruitment of pro-inflammatory monocytes, whereas CCR5 may be involved in the recruitment of regulatory T cells. ELR+ CXC chemokines mediate the recruitment of neutrophils.^6^

**Figure 1:**
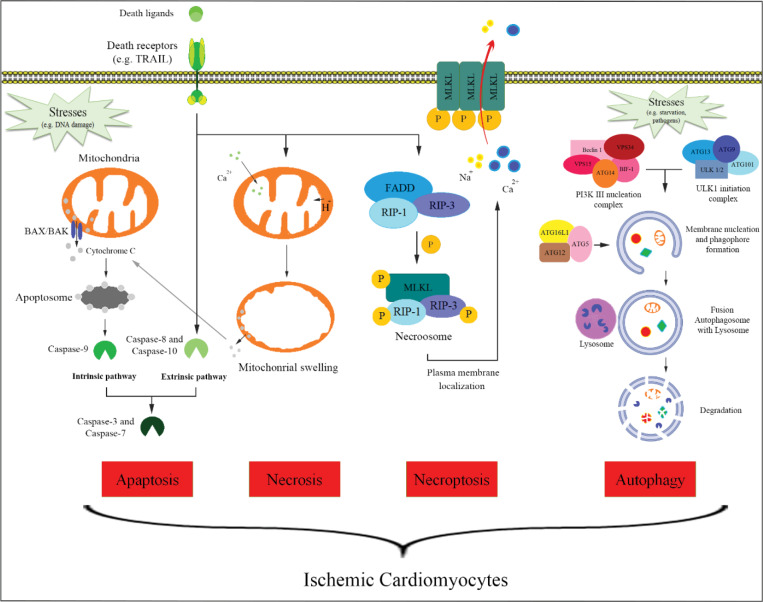
Molecular signaling pathways that involve in ischemic cardiomyocytes

The injury mechanism of CMs result of ischemia includes the apoptosis, necrosis, necroptosis and autophagy.^7^ Apoptosisin the infarcted heart appears to be mediated through the activation of both intrinsic and extrinsic pathways. The crucial event activating CM apoptosis is the permeabilization of OMM (Outer Mitochondrial Membrane), a process that involves interactions between members of the B-cell lymphoma 2 (Bcl-2) family. Ischemia-mediated mitochondrial dysfunction is critically implicated in both necrosis and apoptosis of CMs in the infarcted myocardium.^8^ Necrosis, in contrast to apoptosis, is defined by the opening of the mitochondrial permeability transition pore (mPTP), a pore in the inner mitochondrial membranes. In healthy cells, the inner mitochondrial membrane is impermeable to water, ions, and protons. mPTP opening promotes the influx of water into the mitochondrial matrix, resulting in severe mitochondrial swelling and ultimately triggering necrosis of CMs. cyclophilin D, a prolyl isomerase located within the mitochondrial matrix, is critically involved in mitochondrial swelling and in ischemia-mediated CM necrosis in vitro and in vivo. Mice lacking cyclophilin D exhibit a 40% reduction in infarct size when subjected to reperfused infarction protocols.^9^ Necroptosisis a form of regulated necrosis that could be activated by ligands of death receptors and stimuli that induce the expression of death receptor ligands under apoptotic deficient conditions. Activation of necroptosis by ligands of death receptors requires the kinase activity of RIP1, which mediates the activation of RIP3 and MLKL, two critical downstream mediators of necroptosis.^10^ Initiation of this pathway results in cell swelling, membrane rupture, the release of damage-associated molecular patterns (DAMPs), and inflammation. Blocking the kinase activity of RIP1, a key druggable target in the necroptosis pathway, by necrostatins inhibits the activation of necroptosis and allows cell survival and proliferation in the presence of death receptor ligands.^11^ Recently, studies have been demonstrated that Necroptosis modulates adverse remodeling after MI ^12^ (Figure 1).

In a myocardial ischemic/reperfusion (I/R) model, Nec-1 reduces infarct size, cell damage and neutrophil infiltration, suggesting that Nec-1 effectively mitigates not only ischemic but reperfusion injury.^13^ observations indicate that suppression of necroptosis could be a valuable approach to reduce organ damage caused by hypoxia during infarction. Thus, this novel signaling pathway may be an attractive target for future therapies.^14^

### Underlying mechanisms participating in the cell death after AMI

#### Autophagy in MI

One of the key cellular pathways that mediate stress-induced adaptation and damage control is autophagy. Autophagy, as a pro-survival mechanism that replenishes energy under stress conditions, is activated under MI progression. Ischemia/hypoxia induces autophagy in vivo and in vitro in most investigations,^15,16^ The two pathways responsible for ischemia/hypoxia-induced autophagy involve either BNIP3 ^17^ or AMPK. In a mouse model expressing dominant-negative AMPK in cardiac myocytes, the autophagic response to ischemia was attenuated, leading to larger MI and worse cardiac function ^17^. It seems that autophagy is response in primary stages of ischemia, and if be prolonged, the autophagy becomes dysfunctional.

#### Apoptosis in MI

In the extrinsic pathway, death receptor activation by a death ligand induces death-inducing signaling complex (DISC) formation and casp 8 activation, which in turn activates casp 3. This pathway can also activate the intrinsic pathway by the proteolysis of BID to t-BID by casp 8 and interaction of t-BID with BAX in the mitochondria. Pro-apoptotic BAX/BAK induces cyto c, Smac/DIABLO, AIF, and Endo G release from the mitochondria. Cyto c with Apaf1 and casp 9 form the apoptosome with activation of casp 9. Casp activity is regulated by the endogenous casp inhibitor XIAP.^18^ Cardiac myocytes are naturally resistant to apoptosis due to their low-level expression of Apaf1 and casps and high levels of XIAP.^19^

Also, Bahi *et al* demonstrated that cardiomyocyte levels of all casps decrease with age, and they are very low in adult cardiac cells.^20^ Recently, Bae *et al* reported that apoptosis can be induced in the heart lacking casp activation via casp-independent pathways, probably through apoptosis-inducing factor (AIF).^21^

Mice that lack FAS (lpr mice) exhibited a decrease in cardiac myocyte apoptosis in models of doxorubicin toxicity,^22^ as well as marked reductions in infarct size following I/R.^23^ However, deletion of either TNFR1 or TNFR2 does not affect infarct size. In contrast, deletion of both together resulted in significantly larger infarcts following permanent coronary occlusion.^24^ These results suggest that FAS, and not TNFR, is the major mechanism for activating the extrinsic apoptotic pathway during MI.

#### Necrosis in MI

During certain cardiac pathologies, the action of cellular pumps is inhibited by ATP depletion, there is a consequent increase in H+ and Na+, and the sodium-calcium exchanger operates in reverse manner. Increased cytoplasmic Ca^2+^ leads to increased Ca^2+^ in the mitochondrial matrix along with elevated levels of ROS, culminating in MPTP opening, and necrosis. On the other hand, mitochondrial swelling and mitochondrial membrane rupture also produce necrosis. Moreover, increased H+ in the cytoplasm and inactivation of H+ pumps elicit declines in lysosomal pH, which results in overactivation of proteases such as cathepsins. The massive entry of water results in lysosomal swelling, membrane rupture, and release of proteases into the cytoplasm, which together with other activated proteases, such as calpains, digest different substrates, including cytoskeletal proteins, contributing to necrosis. Activation of death receptors, such as the TNF-αreceptor, represents other necrosis pathways in cardiac myocytes under certain conditions such as HF. The activation of these receptors could lead to the activation of receptor-interacting protein (RIP), increased ROS, and necrosis. The massive inflow of water into the cell by the osmotic imbalance ultimately leads to cell swelling and rupture of the plasma membrane.^24^

### Normal & abnormal healing process of MI

The phases of Normal repair following MI including Inflammatory, Proliferation and Maturation. DAMPs (Alarmins) released by necrotic CMs trigger an intense inflammatory reaction that serves to clear the infarct from dead cells and matrix debris. Removal of dead cells induces suppression of pro-inflammatory signaling, leading to the transition to the proliferative phase. In this phase, Fibroblasts can acquire one of the myofibroblasts, proliferative and matrix synthetic phenotype and then, deposit extracellular matrix (ECM) proteins and a rich neovascular network is formed.^25^ Finally, during the maturation phase, the ECM is cross-linked, while infarct fibroblasts become quiescent and may undergo apoptosis. Well-healed infarcts contain large amounts of ECM that can occupy up to 80% of the infarct area. However, collagen deposition also occurs in the un-infarcted remote myocardial region, predominantly in the interstitium, where it contributes to ventricular stiffness and dysfunction. Myocardial interstitial fibrosis directly contributes to adverse structural remodeling in various CVDs that are associated with chronic ischemia and or pressure overload, as well as in some intrinsic myocardial diseases, such as hypertrophic and diabetic cardiomyopathy. In addition to interstitial alterations in the noninjured areas, replacement fibrosis, although it initially supports ventricular morphology after MI, can contribute to geometric changes and inevitable functional deterioration over time. The granulation tissue post-MI consists of inflammatory cells, neovascularization and fibroblast-like cells that deposit collagen. The granulation tissue matures into a scar. Adequately healed infarcts show preserved geometry, while inadequately healed infarcts with few myofibroblasts show severe dilatation and infarct expansion. Therefore, an optimally healed infarcted heart should comprise an ECM-rich replacement scar but minimal remote fibrosis.

### Current Therapies of MI

Surgical operations are sometimes required to treat CVDs. They include: medical therapy, coronary artery bypass grafting (CABG), and other costly surgeries.

### Cell Therapy in MI

Because of difficulties, outcomes and backwashes of traditional therapies of MI, the novel strategy in the treatment for ischemic cardiomyopathy after MI, cell therapy, newly emerging. An appropriate regenerative strategy of cell population is critical for operative cell therapy and has been documented in many researches.

### Cell source used in myocardial Stem Cell therapy

Perhaps the most stunning aspect of current progress towards cardiac regeneration is the wide variety of cell types that have been considered as candidates for therapeutic delivery in humans. Numerous pre-clinical and clinical studies have assessed a variety of cell types for cardiac regeneration. Different types of stem cell and progenitor cell have been shown to improve cardiac function through various mechanisms, including the formation of new myocytes, ECs and vascular smooth muscle cells, as well as through paracrine effects. From the histological view, these cells divided into two cluster; one group differentiate into cardiac cells and another group make supportive tissue for cardiac cells, including bone marrow- and/or adipose tissue-derived mesenchymal stem cells (MSCs), embryonic stem cells (ESCs), autologous skeletal myoblasts, induced pluripotent stem cells (iPSC), endothelial progenitor cells (EPCs), cardiac progenitor cells (CPCs) and cardiac pericytes (CPs), newly emerging cell source for cardiac stem cell therapy. The majority of these cell populations are produced pre-clinically and are safe and effective in clinical practice.^26^ This numerous cell types reflects the unmet medical need for treating heart disease, and hence the large amount of experimental effort being put into devising cell-based therapies. As shown in Table 1, In the next section, any cell type used in cardiac stem cell therapy briefly described:

**Table 1:** Supportive tissue for ischemia cardiac cells

**Type**	**Inherent property**	**investigations**	**Source for separation**	**Ref**
Mesenchymal Stem Cells (MSCs)	Increase angiogenesis and repair tissue damages, differentiate to mesenchymal lineage, adhesive properties to vascular endothelium surface	Adding in culture of Mouse mesenchymal stem cells and human ECs commence to formation tube like structures in Matrigel immediately	Bone marrow, peripheral blood, adipose tissue, placenta, umbilical cord, cord blood	15-20
Endothelial Progenitor Cells (EPCs)	Increasing blood circulation endothelial progenitor cells, Improvement and development of neovascularization in tissues	Improving ischemic organs’ function with induct angiogenesis or rebuilding of damaged blood vessels	Adult bone marrow, peripheral blood, cord blood, stationary cells in tissue like human stem cells	22-24
Embryonic Stem Cells (ESCs)	Differentiate to any linage of cell lines self- inclusive germ line after injection into fibroblasts in cell culture	Replacement with death cells in the different tissues that body has not able to replace	From the inner cell mass of the blastocyst	29-33
Induced pluripotent Stem cells (iPCs)	Differentiate to any linage of cell lines self- inclusive germ line after injection into fibroblasts in cell culture	Transplanting into CMs in vitro resulting in improved function of damaged tissue	blood circulation	25-28
Skeletal myoblasts	Able to expand and proliferate after isolating, resistant against heart attacks and ischemia	increasing of small vessel formation in one of three patients, differentiated skeletal muscle cells into mature myofibers	Generated by tissue-resident stem cells termed “satellite cells” or muscle stem cells (MuSCs)	34,35
Cardiac Progenitor Cells (CPCs)	Self- renewal and differentiation	Following MI the number of CPCs/sca-1+ increase that may participate in repair damaged cells	Existence in atrium and vertical as 1 percent of heart cells	36-38
Scaffolds	A structural platform for a new cellular microenvironment, mimic cardiac tissue flexibility, allow sufficient oxygen and nutrient delivery to cells and allow physiological electrical propagation	Natural polymer- based scaffold with more biocompatible, less immunogenic, higher capacity for cell adhesion but their limited mechanical and biodegradable properties, synthetic polymer-based that easily tailored with predictable mechanical and chemical properties but their inflammatory response	Natural polymer collagen, fibrin, alginate, Matrigel, chitosan and hyaluronic acid synthetic polymer polyethylene glycol (PEG), poly hydroxyl ethyl methacrylate, polylactide-glycolic acid (PLGA) and poly (N-isopropyl acrylamide)	39
Pericytes (PCs)	Differentiate to different type of cells, high ability to change to fibroblast cells, regulate vascular remodeling and control proliferation and differentiation of ECs, provide vessels stabilization and maturation	Microvascular formation inside the myocardium in response to hypoxia, exhibit cardiomyogenic potential in in vivo	Surround of ECs in small blood vessels	40-42,49

### Mesenchymal Stem Cells (MSCs)

In recent years stem cells particular MSCs declared as a source to increase angiogenesis and repair tissue damages .^27^ By addition these cells have pluripotent progenitor trait and cause making different parts of skeletal tissue: ligament – marrow stroma – cartilage and connective tissue.^28^ The number of specific intrinsic and extrinsic factors lead MSCs to particular pathway to express molecular and cellular patterns in a specific tissue. Also MSCs have adhesive properties to vascular endothelium surface through external inflammation mechanisms.^29^ Current cells adhere to the endothelium through having interaction with integrin, so this interaction lead to neovascularization in ECs. MSCs have high capacity to differentiate to mesenchymal lineage like adipose tissue and chondrocytes and have the ability to dividing into any type of cells.^30,31^ Moreover, Adhesion to plastic culture dishes and other plastic veins is other properties of MSCs .^32^ Likewise, these cells have self-renewal properties and they were shown to express cell surface markers like CD44, CD29, CD73, and CD73 except CD45 and CD34.^33^ Mouse mesenchymal stem cells and human ECs were cultured in Matrigel at the same time.^34^ The result showed, after adding MSCs and ESCs, they commence to formation tube-like structures in Matrigel immediately.

### Endothelial Progenitor Cells (EPCs)

In 1932 reports proved blood circulation EPCs increasing, cause an increase of mature ECs.^35^ Differentiation and self-renewal properties of EPCs established a great hope to the usage of this cell population in regenerative medicine and cardiac infarction treatments. For the first time EPCs in the blood circulation were separated and described by *Ashara* and colleagues. They separated CD34+ cells from peripheral blood by using magnetic beads and cultured them. Cultured cells had properties similar to ECs under specific conditions. Adult bone marrow – peripheral blood – cord blood – stationary cells in tissue-like human stem cells, are as a source of ECs.^35^ EPCs from bone – marrow with angioblastic properties improve ischemic organs’ function with inducing angiogenesis mediators in low oxygen places or with inducing rebuilding of damaged blood vessel ECs.^36^ Generally, there are different sorts of EPCs, but based on the cell sources hematopoietic and non-hematopoietic EPCs have been isolated.^37^ In the term of cell surface markers, discovered markers are not exclusively belonging to the EPCs and there are some common surface markers between mature ECs, hematopoietic and endothelial progenitor cells. The precursor and specific cell surface markers of EPCs have been remained unclear. Vice versa, data from recent studies elucidated that circulating EPCs has been enriched in CD34, CD133 and VEGFR2, vascular endothelial cadherin and, CD31, which are potential to use in the treatment of the ischemic condition such as myocardial infarction in mice. On the other hand, CD34+/ CD133+ /VEGFR2+ cultivated cells have differentiation ability into hematopoietic and not endothelial lineages,^38^ these findings support this idea that the clonal cultivated cells were belong to non-angioblastic hematopoietic progenitors which apply their angiogenesis properties by using paracrine effects. In the same study, it has been reported that CD34+/CD45- cells from the non-hematopoietic population differentiated to mature endothelial cells, which had the ability to participate in the formation of vessel-like structures.^39,40^ The mobilization, recruitment and circulation potency of the EPCs under the ischemic condition to sites of neovascularization should not be neglected. Previous data also suggested the crucial role of the Tie-2 receptor as a surface marker of EPCs in the development of angiogenesis. To addresses these findings there is an experiment, which highlighted the crucial role of Ang-1 and Ang-2 as ligands of Tie-2 on the vessel formation properties of EPCs. The results showed significant increasing of the tubulogenic potential of EPCs in Matrigel assay after incubation with Ang-1 .^41^ Of note, whether EPCs appreciate the participation to neovascularization and angiogenesis based on their differentiation, self-renewal, and cytokine secretion properties, application of mentioned cells in the field of regenerative medicine need further investigations to overcome the presence pitfalls and challenges.

### Induced pluripotent Stem cells (iPCs)

In regenerative medicine iPCs as important pluripotent stem cells from adult cells.^42^ The IPCs are very similar to ESCs in the pattern of chromatin methylation and express of some certain genes and proteins.^43^ Research had shown mature cells can be reprogrammed to pluripotent stem cells with reprogramming their genes.^44^ Some specific transcription genes to achieve desired results and using mature cells as pluripotent cells to increase their differentiation in regenerative medicine including Oct4, Sox2, c-Myc, and KLf4.^45^ Indeed, those factors are as original reprogramming factors in mature cells. However, the rate of regeneration and deviation of IPCs is very low and ineffective as takes 3-4 weeks or 1-2 weeks in human and mouse cells respectively, so the efficiency of about 0.01-1 percent. Nevertheless, iPCs have tumorigenesis properties, but a study on mice has not shown any effects of tumorigeneses. Hence to solve this obstacle, before injection iPCs to infarct tissue as well as infarct heart they had transplanted into CMs in vitro. This transplantation was successful, cells were localized in cardiac and the function of damaged tissue had improved.

### Embryonic Stem Cells (ESCs)

ESCs have pluripotent properties and they can differentiate to any linage of cell lines self- inclusive germline after injection into fibroblasts in cell culture.^46^ They had preserved pluripotency of themselves at the same time and differentiated onto a hundred number of specific cell lines.^47^ On the other hand, ESCs replace with dead cells in the different tissues of the human body, in some diseases instance heart attack, stroke, and blindness that body has not more ability to replace papulation of losing cells.^48,49^ but the major problem with using them in regenerative medicine as therapeutic cells are their uncontrollable totipotent properties of these cells.^50^ indeed, the plasticity of ESCs make them difficult to control, for this reason, they should be maintained in the undifferentiated stage to use them as therapeutic cells in regenerative medicine.

### Skeletal myoblasts

Skeletal myoblasts are one the sufficient cells can use in cardiac regenerative medicine as progenitor cells that they have a high capacity of myogenic properties.^51^ Also, these cells were found with high exuberance in the human body. Skeletal myoblasts have two important ability including able to expand and proliferate after isolating and resistant against heart attacks and ischemia.^52^ In 2003, *Pagani* and colleagues had shown that transplantation of myoblasts to scarred myocardium is conceivable. They were transplanted differentiated skeletal muscle cells into mature myofibers. Results had confirmed increasing of small vessel formation in one of three patients.^53^

### Cardiac Progenitor Cells (CPCs)

CPCs are one of the important cells with self- renewal, differentiation, and pluripotency properties that they can use in regenerative medicine as therapeutic cells.^54,55^ These cells exist in atrium and vertical as 1 percent of heart cells.^56^ Research had shown these cells express sca-1 and c-kit of cell surface markers.^56,57^ In 2003, *Hidemasa Oh* and colleagues had shown the existence of stem cell markers in CPCs. They isolate two type of CPCs with sca-1+ and sca-1- from mouse myocardium. Likewise, they proved self- renewal reactivity related to telomerase reverse transcriptase in sca-1+ cells. Following MI, the number of sca-1+/CD31- cells increased, they may participate in repair damaged cells in MI respectively. It is clear that the number of stem cells and other pluripotent cells will increase after receiving stimulation signals from infarcted myocardium in bone marrow but research and experiments by FACS technology proved no symptoms of increasing CPCs in the bone marrow, it may show that increasing of CPCs in left atrium vertical after MI is absolutely endogenous in the heart.^58^

### Pericytes (PCs)

PCs (also known as Rouget cells and mural cells) are a kind of perivascular cells with pluripotent and differentiation capacity and expressing cell surface markers include: CD75, CD90, and CD105 like mesenchymal stem cells. In comparison with ECs they cannot express CD34, CD45, CD31, and von Willebrand Factor (vWF) of cell surface markers don’t express endothelial or hematopoietic cell surface antigens such as CD31, CD34, CD45, and vWf. Likewise, they can differentiate to different cell types include osteogenic, adipogenic, vascular smooth, and skeletal muscle cells, also in inflammatory conditions these cells have a high ability to change to fibroblast cells with collagen Type-I producing properties. Other significant traits of PCs is that they express macrophages markers like CR3 complement receptor, CD4, and class I and II major histocompatibility (MHC), so they can act as phagocytes. One of the excellent criteria of this population of stem cells is that they have a large number in cardiac tissue and the most resident stem cell of the heart. Moreover, these cells are immunogenically safe and not an initiate immune reaction when used in stem cell therapy. The PCs are located in the surround of ECs in small blood vessels and also among of tunica intima and media intima in large vessels with peg-and-socket contacts. Recently PCs have become as important cell sources for tissue engineering and regenerative medicine in cardiac regeneration.

### Mechanism of action of PCs in cardiac tissue regeneration

PCs, by direct (peg-and-socket contacts) and indirect ways (signaling pathways and paracrine mechanisms) regulate vascular remodeling. They control proliferation and differentiation of ECs (through TβR/ALK- 5/Smad2-3 pathway), provides vessel stabilization and maturation (by Ang1/Tie2 and Ang2/Tie2 pathways) and migrate along vessel through angiogenesis (via PDGF-β/PDGFR-β signaling). It has been reported that PCs are one of potential sources of fibroblasts^59^ and can differentiate into cardiac myofibroblasts types during inflammation.^60^ During later phases of infarct healing, PCs, and smooth muscle cells interact via PDGF and PDGF receptor-β signaling, resulting in neovessels maturation and covered a mural coat.^61^ In line this, it has been demonstrated that early neovessels in the infarcted heart are pro-inflammatory and hyperpermeable, lacking a PC coat.^62^ The acquisition of a PC coat reduces vascular permeability and angiogenic potential of the vessels, contributing to the formation of a stable scar. Therefore, PC coating is an important stage for suppression of granulation tissue formation following MI, and improve the resolution of inflammation and stabilization of the scar.

PCs, also, because of their secretome, have an immunomodulatory and cardio-protective effect. In the paracrine function of PCs, studies documented that PCs secrete several factors such as chemokines and cytokines that participate in inflammatory responses modulation. Studies demonstrate that PCs overexpress IL- 6, LIF, TGF-b1, COX-2, HMOX-1, and HIF-1a, which are sustained under hypoxic conditions. However, MCP-1, IL-4, IL-10, iNOS, 2,3-IDO, pro-inflammatory cytokines expression including IL-1a, TNF-a, and IFNc, dramatically diminished under hypoxia, suggesting an immune-regulatory cytokine secretome that is exclusive to human vascular PCs.^63-66^ *Cheryl et al*declare that PCs are safe as a point of immunogenic reaction and modulate CD4 positive T cell responses.^67^

During vasculogenesis and angiogenesis PCs participate in the maturation and stabilization of the vascular system and transfer angiogenic factors. Of late, PCs have emerged as a new cell source for tissue engineering applications .^68^ Their function of supporting vascular network, vessel maturation/stabilization, and angiogenesis, makes PCs an attractive cell source of stem cell-based tissue-engineered heart regeneration approaches. It has been shown in many of vascular tissue engineering investigation that co-culture of PCs from different sources with HUVECs results in basement membrane formation, developing endothelial sprouts, microvascular networks growth, In vitro pre-vascularization, tubule formation and increase vessel density in both of the peri-infarct collateral circulations and within the infarct region.^69^ Moreover, it has been demonstrated that PCs expression of ANG-1, activates Tie2 receptors in both PC and EC and triggering downstream pathways that participate in vascular maturation.^70^

PCs, compared to other types of stem cells, appear to well-engraftment in the infarcted myocardium, probably attributable to several factors. The increased proliferation and migration of PCs in response to low oxygen concentration and ECM degradation products have important implications for ischemic injury repair. The perivascular niche-homing capacity may further benefit the long-term survival of PCs. The potential of human muscle PCs to reconstitute major cell types in the injured myocardium was hereby demonstrated. Cell fate tracking suggests that a minor fraction of donor PCs differentiated into and/or fused with CMs.

**Differentiation**. PCs, like MSCs, are a multipotent stem cell type. They have the potency to transdifferentiate into the mesenchymal lineage such as myocytes. This ability of PCs may contribute to regenerative medicine application following cardiac tissue injury or cardiovascular disease .^71^ In line with, it is well-known that PCs demonstrate an ischemia/hypoxia-induced response. Moreover, it has been confirmed that microvascular PCs inside the myocardium demonstrate angiogenic behavior in response to hypoxia and exhibit cardiomyogenic potential in *in vivo.*^72^ This opens novel and promising prospects for transplanting autologous PCs within the infarcted heart, and be applicable cardiac regenerative medicine.

*Katare R et al* demonstrated that human PCs transplantation promotes the repair of the infarcted heart through activation of an angiogenic process involving micro-RNA-132.^73^ Acquisition of a PCs coat by angiogenic vessels in the infarcted heart might suppress inflammatory activity stabilizing the microvasculature and preventing prolonged recruitment of leukocytes.^74^

*Avolio et al* first described the relationship of the PCs with the endogenous cardiac stem cells and reporting that co-transplantation of both cells reduce dramatically scar size in mouse infarcted heart.^75^ Furthermore, PCs can repair tissue by trophic factors secretion.^76^ Chen et al well documented that transplantation of PCs contributes to the functional and structural repair of the ischemic heart through paracrine effects and cellular interactions. Moreover, PC therapy prevents progressive dilatation of left ventricle (LV) and heart failure and attenuate of deleterious myocardium remodeling. Besides, their team demonstrated significant improvement in cardiac contractility in an acute infarction milieu. The therapeutic benefits observed could be explained, by anti-fibrotic, anti-inflammatory, angiogenic, and to an extent, cardiomyogenic properties of PCs.^77^ They showed myocardial fibrosis significantly reduced following PCs transplantation. Along with the attenuation of progressive LV dilatation, PCs treatment appears to result in propitious remodeling, leading to improved myocardial compliance and strengthening of the ischemic cardiac tissue.

In this study, PCs treatment significantly diminished host infiltration of monocyte/macrophage in the infarcted myocardium and proposing an anti-inflammatory potential which contributed to the reduction of fibrosis, amelioration of adverse remodeling, and improvement of cardiac function.

### Scaffolds used in Cardiac Regeneration

A “scaffold” is a substitute that provides a structural platform for a new cellular microenvironment that supports new tissue formation. The scaffold used in Cardiac Regeneration should be Non-toxic, Non-immunogenic, Biodegradable and degradation rate should match the rate of native ECM replacement, mimic cardiac tissue flexibility, allow sufficient oxygen and nutrient delivery to cells, and allow physiological electrical propagation. Totally, Scaffolds used in Cardiac Regeneration divided into two groups: 1) Natural polymer-based scaffolds such as collagen, fibrin, alginate, Matrigel, chitosan and hyaluronic acid that biodegradable, more biocompatible, less immunogenic, higher capacity for cell adhesion but their limited mechanical and biodegradable properties are not easily tailored. 2) synthetic polymer-based scaffolds such as polyethylene glycol (PEG), poly hydroxyl ethyl methacrylate, polylactide-glycolic acid (PLGA) and poly (N-isopropyl acrylamide) that easily tailored with predictable mechanical and chemical properties but they may elicit an inflammatory response. Natural polymer-based scaffold or natural polymers used in clinical trials as biodegradable materials at the first. These scaffolds have bioactive properties and they have better interactions with cells that increase repairability of these compounds in biological systems. Natural polymers are including polysaccharides, proteins, and polynucleotides. Despite the compatibility of natural polymers with biological systems and damaged body because of their belonging to different parts of the body (hyaluronic acid can produce from extracellular tissue), synthetic polymers are useful than natural polymers because they can be produced for specific applications bases their degradation time and mechanical characteristics. Also, they are cheaper than natural polymers and produce on large scales. Some of the synthetic polymers that using in tissue engineering are including PLA, PGA and PLGA. Whereas PHA is one of the microbial polyesters that it is using in tissue engineering, increasingly.

### Scaffold-free constructs

The low cellular concentration inside the scaffold, induce inflammatory reaction and fibrous tissue formation caused by scaffold degradation, led to the consideration of building tissue engineering constructs without the use of scaffolds. Cell sheet technology is an attractive alternative for treating advanced MI that has progressed significantly over the past two decades. Myocardial cell sheet (MCS) technology enables the construction of myocardial tissue with correctly aligned CMs. Cell sheets have a two different type: Fibrin-based Cell Sheets and Temperature responsive-based Cell sheets. In Fibrin-based cell sheets, Culture dishes were coated with fibrinogen monomers mixed with thrombin and stored at 4°C. CMs were seeded onto the fibrin polymer, which was then degraded within 4 days by proteases secreted from the CMs. The MCS could be detached from the dish with a cell scraper. When these MCSs were overlapped, they connected to each other through connexin-43 and conducted action potentials. MCSs generated using this method and transplanted onto subcutaneous areas of nude rats beat spontaneously. Temperature-sensitive culture dishes were created by grafting temperature-responsive polymer (poly-N-isopropyl acrylamide) onto the surface of the dish. At 37°C, the polymer surface is hydrophobic and cells can attach onto the dishes. When the temperature is dropped below 32°C, the surface becomes hydrophilic and the grafted polymer rapidly hydrates, making it expand and causing the cells to detach from the surface. When compared with enzymatic digestion of a scaffold, which often disrupts the cell-cell contacts, this innovative technology enables a cell sheet to degenerated just by cooling the dishes to room temperature and using multiple layered cell sheets.^78^

### The role of EPCs in CVDs

EPCs were purified from various sources of a human body such as cord blood, bone marrow and peripheral blood mononuclear cells that express hematopoietic stem cells surface markers like, CD 133 (it is absent in mature ECs) and low level of CD34.^79^ Also, they are positive for endothelial surface markers like VEGFR2, vWf, and ability for differentiate to mature endothelial lineage as basic cells in angiogenesis, vascularization and neo-vascularization process in inflammatory and cardiovascular diseases. Generally, shaded ECs from vessel walls express CD34 and endothelial progenitor cells are positive for CD133 and either express VEGFR2.^80,81^ Studies had shown, the EPCs and stem progenitor cells expanded in *in-vitro* condition improved cardiac function and reduced the scarring of left ventricular in animal models.^82^ On the other hands, the EPCs which were isolated from peripheral blood significantly increased vascularization and neovascularization in the ischemic tissue after migration and homing to rescue tissue.^83^ Indeed, it is demonstrated that cultivation of endothelial progenitor cells from various sources after cardiac infarction and inflammatory diseases, EPCs released to peripheral blood and expressed high levels of cytokine receptors as VEGF, HGF and, IGF-1 that activation of these receptors by released cytokines from microphages/monocytes cause migration of EPCs to ischemic tissue.^84^ Additionally, EPCs could incorporate into newly forming vessels in re-endothelialization and neo-vascularization process.^85^ It has been speculated that studies also had shown the levels of SDF-1 proteins as chemotactic factors were increased after induction of heart attack and cardiac infarction.^86^ Indeed, during the first day after infarction, SDF-1 increased migration of stem and endothelial progenitor cells to ischemic tissue and incorporation of them in re-endothelialization. Also, VEGF has acted as a chemotactic factor to migration of EPCs but surprisingly hematopoietic stem cells didn’t sensitive to G-CSF,^87^ so it seems that G-CSF couldn’t act as a chemotactic factor for EPCs.^86^ The mobilization of stem cells from bone marrow in inflammatory diseases has depending on stem cell microenvironment of stem cells called “stem cells niche” that consist of ECs, fibroblasts, and osteoblasts.^88^ In this era of Bone- marrow stem cells have structural interactions with stromal cells that mobilizing cytokines in an inflammatory condition and cardiovascular diseases inhibit from establishments of this connectors through activation of some important proteinases like matrix-metaloproteinase-9 (MMP-9), elastase and cathepsin-G that cleave adhesive bonds between stromal cells and integrins on hematopoietic stem cells.^89,90^ Overall in ischemic condition mobilization of EPCs induced by VEGF and SDF-1 factors release in ischemia to blood flow and stimulate the mobilization of endothelial progenitor cells from Bone marrow through MMP-9 mechanisms that have the ability to cleave membrane-bound kit ligand (mKitL) and induce the releasing of endothelial progenitor cells to blood circulation to participate in the rescue of damaged tissue or ischemia. More investigations were shown that endothelial progenitor cells are positive to expressing SDF-1 and HGF and IGF-1 also the granulocyte colony stimulating factor (G-CSF). On the other hand, they have shown these cells release growth factors to condition media on neighboring cells to stimulate angiogenesis, for instance, VEGF, SDF-1, IGF-1that paly a basic role in stimulate angiogenesis and rescue damaged tissue for example in MI.^91,92^ This studies approve that the supernatant of EPC’s condition media significantly were increased the migration of mature ECs and CPCs to damaged tissues. It has worthy of note that VEGF is one of the basic pro-angiogenic factors to stimulate differentiation, migration and maturation of EPCs in inflammatory situations from bone marrow that the mechanism of releasing by recruitment of SDF and its interaction with MMPs was described as mentioned previously.^93,94^ However, encouraging results with strong evidence had shown that the EPCs mobilize into circulating exactly in either patients with acute coronary disorders and acute myocardial infarction, but not in stable myocardial infarction.^94^ Likewise, plus more supporting data regard to mobilizing of EPCs in ischemic condition, so concerning issues have been highlighted related to the growth factors and molecular pathway mechanisms of EPCs migration, maturation and also differentiation into ECs.

Currently, more than 150 studies have been done regarding to regenerative medicine applications of EPCs in clinical trials, so obviously the applications of EPCs in vascular regenerative should not be overlooked in myocardial infarction and ischemic diseases. With regard to current and completed studies have identified tree major applications of EPCs: EPCs mobilization therapies, EPCs capture stents, and cellular injection for ischemic diseases. EPCs are as a great cell source because of their ready accessibility from peripheral blood. Potential ability to vasculogenesis and also a low risk to tumorigenesis. In the study regard to clinical trial usage of EPCs was shown that after injection of EPCs tissue perfusion significantly was improved in salvage rats.

Capture stents separate EPCs from blood circulation to promote re-endothelialization of the luminal surface. In this avenue, immobilized antibodies connected to stent surface specially and typically against CD34. The regenerated endothelia are a candidate to diminish the risk of stent thrombosis, restenosis and also need for prolonged anti-coagulative regimens. On the other hand, conjugation of antibodies causes surface passivating and reducing platelet adhesion and also has coagulative effects to increase homo-compatibility measures. In exogenous mobilization, circulating EPCs mobilized and conduced to ischemic tissue by utilizing of cytokine therapy. Following cytokine therapy, the increased EPCs in circulating then stimulated to migrate and homing to the ischemic site to augmentation of neo-vascularization and re-endothelialization. The use of exogenous mobilization obviously is easy to use in clinical trials because of external manipulating elimination and additionally drug-using protocols are so safe and have been established very well.

### Clinical trials in cardiac regenerative medicine

As described previously, stem cells appreciated to have proliferation, differentiation, self-renewal, and other prominent properties, which authorized them to be one of the noticeable candidates in cardiac regeneration therapy. There are various randomized clinical trials in the utilization of autologous bone-marrow isolated MNCs in the context of regeneration medicine in heart failure. The promising results were revealed the significant improvement of the infarcted zone in phase I/II clinical trials.

To address the utilization of cell therapy in the AMI, there are evidences, which highlighted the focus of human umbilical cord blood-derived mononuclear cells (UCB-MNC) in the field of regenerative medicine. The mentioned cells have the potential ability to differentiate to various and specialized cell types in the term of cardiac infarction regeneration. There is one study, which surveyed the feasibility and safety of direct injection of these UCB cells to the intramyocardial in infants with hypoplastic left heart syndrome (HLHS). It has been reported that the initial injection of UCB-MNC in 10 patients improved the cardiac function after follow up during 3 and 6 months .^95^ Additionally, there is another study, which investigated the role of CD133+ stem cells in ischemic cardiomyopathy during coronary artery bypass grafting. In the current study, up to 10 million of CD45, CD133, CD34 bone-marrow isolated cells were injected to the infarcted zone in 7 patients and the results were analyzed after 6 months follow up. The results of magnetic resonance imaging elucidated the improvement of left ventricular volumes in all injected patients.^96^ The clinical trial phase of cardiac regeneration medicine has not been stopped in phase I/II clinical trials. In a study, the randomized phase III of the clinical trial have been investigated in the myocardial infarction after intramyocardial application of bone-marrow isolated CD113+ in 82 patients with chronic ischemia and decreased Left ventricular ejection fraction (LEVF). In the current study, the participants were organized into two groups, which 5 ml of placebo and suspension of 0.5–5 × 10^6^ CD133^+^ cells were injected to each group as a control and treatment group subsequently. Findings showed the improvement of the cardiac infarction zone in the participants. Besides, data analysis elucidated the significant tie between induced cardiac repair and different circulating cells such as CD133^+^ EPC and thrombocytes.^97^

## Discussion

The natural process of improving MI is very slow, accompanied by inflammatory reactions and often abortive. On the other hand, traditional remedies such as transplant procedures are very expensive and have many complications. Therefore, novel approaches for the treatment of MI are important. Cell therapy is an emerging way in which a cell population is created efficiently to treat a variety of diseases caused by cell death. Various sources have been used as a cellular source for the treatment and regeneration of the heart. Studies have shown that EPCs, MSCs, ESCs, autologous skeletal myoblasts, iPSC, PCs, and many other stem cells are used as cellular sources for cell therapy due to their high proliferation and differentiation potential. The scaffolds, due to their proper interaction with the cells, provide a suitable environment for cell growth and new tissue formation. Today, these scaffolds are used as an extracellular substrate for the growth of heart cells. PCs cells are high-proliferation and differentiating vascular cells that function as phagocytes in addition to repairing damaged heart tissue. These cells are not immunogenic and have the potential to differentiate into cardiac myofibroblasts. PCs also reduce the permeability of the vessel wall and increase angiogenesis. PCs repair the heart by activating downstream angiogenesis and differentiation to myofibroblasts.

## Conclusion

In general, cell therapy is one of the new and highly effective methods in the treatment of cardiovascular disease that has great potential for development and epidemic.

## Acknowledgements

We thank the Department of Clinical Biochemistry and Laboratory Medicine academic members for their valuable assistance in preparing this article.

## Competing interest

The author(s) declare(s) that there is no conflict of interest regarding the publication of this paper.

## Funding

None.
